# Circular RNA circ_0057558 Controls Prostate Cancer Cell Proliferation Through Regulating miR-206/USP33/c-Myc Axis

**DOI:** 10.3389/fcell.2021.644397

**Published:** 2021-02-26

**Authors:** Tao Ding, Yanjun Zhu, Huimin Jin, Ping Zhang, Jianming Guo, Jianghua Zheng

**Affiliations:** ^1^Department of Urology, The Sixth People’s Hospital South Campus, Shanghai, China; ^2^Department of Urology, Zhongshan Hospital, Fudan University, Shanghai, China; ^3^Department of Laboratory Medicine, Zhoupu Hospital Affiliated to Shanghai University of Medicine and Health Sciences, Shanghai, China

**Keywords:** prostate cancer, proliferation, deubiquitination, sponge, circular RNA

## Abstract

We previously reported the elevated expression of circ_0057558 in prostate cancer tissues and cell lines. Here, we aimed to determine the biological function of circ_0057558 in prostate cancer. In the current study, circ_0057558 knockdown in prostate cancer cells significantly repressed cell proliferation and colony formation, but promoted cell arrest and enhanced the sensitivity to docetaxel. Bioinformatics analysis prediction and RNA-pull down assay identified miR-206 as the potential binding miRNA of circ_0057558. A negative correlation was observed between the expression of miR-206 and circ_0057558 in prostate cancer tissues. miR-206 mimics rescued the function of circ_0057558 overexpression on prostate cancer cells. Further, the bioinformatics analysis and luciferase assay suggested that miR-206 may target ubiquitin-specific peptidase 33 (USP33). USP33 mRNA expression has negative correlation with miR-206 expression and positive correlation with circ_0057558 expression in prostate cancer tissues. USP33 overexpression partially blocked the effects of miR-206 mimics on prostate cell proliferation. USP33 could bind and deubiquitinate c-Myc. Increased c-Myc protein by circ_0057558 overexpression was partially reversed by miR-206 mimics. The proliferation inhibition activity of MYC inhibitor 361 (MYCi361) was more prominent in primary prostate cancer cells and patient-derived xenograft (PDX) model with higher level of circ_0057558. Collectively, circ_0057558 gives an impetus to cell proliferation and cell cycle control in prostate cancer cell lines by sponging miR-206 and positively regulating the transcription of the miR-206 target gene USP33.

## Introduction

Prostate cancer is one of the most frequent malignancies in men, causing high morbidity and mortality (Siegel et al., 2016). Currently, prostate-specific antigen (PSA) testing is the standard screening marker for prostate cancer diagnosis. However, because of the poor specificity of PSA screening, it contributes to overdiagnosis and subsequent overtreatment (Tarhan et al., 2005; De Nunzio et al., 2011). Currently, docetaxel represents the most active chemotherapeutic agent for prostate cancer. However, inherent or acquired drug resistance limits the efficacy of docetaxel (O’Neill et al., 2011). Therefore, it is urgent to identify novel diagnostic and prognostic biomarkers for prostate cancer and improve our understanding of the molecular basis of prostate cancer.

Circular RNAs (circRNAs), discovered as a non-coding RNAs (Sanger et al., 1976; Cocquerelle et al., 1993), are conserved between different species (Qu et al., 2015; Chen, 2016). circRNAs form closed-loop structures without cap-structure and poly(A)-tail. Due to such structural properties, circRNAs is resistant to the digestion of exonucleases and more stable than linear RNA. Similar to the long non-coding RNAs (lncRNAs), circRNA could absorb target microRNAs (miRNAs), thus regulating the transcription of genes targeted by aforementioned miRNAs (Qu et al., 2015; Chen, 2016). Recent reports have identified circRNAs as novel diagnosis markers for diverse cancers, such as hepatocellular carcinoma, esophageal squamous cell carcinoma, gastric cancer, and bladder cancer (Xia et al., 2016; Zhong et al., 2016; Fu et al., 2017; Lu et al., 2017). Evidence has supported the roles of circRNAs in the process of cancer cell proliferation, apoptosis, and invasion through acting as sponges for miRNAs. For instance, CDR1as/ciRS-7 targets miR-7 to regulate proliferation in the development of hepatocellular carcinoma, colon cancer, and gastric cancer (Tang et al., 2017; Xu et al., 2017; Pan et al., 2018). Overexpression of circCHIPK3 inhibits the expression of miR-124 and increases the expression of miR-124 target gene, interleukin 6 receptor (IL6R), which leads to cancer cell proliferation (Zheng et al., 2016). circ_0013958 can enhance lung adenocarcinoma cell proliferation by competitively binding miR-134 and upregulating cyclin D1 (Zhu et al., 2017). Previously, we have identified that circ_0057558 expression was elevated in prostate cancer tissues and may serve as a novel biomarker for prostate cancer using human competing endogenous RNAs (ceRNA) microarray (Xia et al., 2018). However, the functions of circ_0057558 in prostate carcinogenesis remain largely unknown.

In the present study, we conducted *in vitro* and *in vivo* experiments to unravel the biological functions of circ_0057558 in prostate cancer cell proliferation, cell cycle transition, and docetaxel resistance. Further, we found that circ_0057558 could sequester miR-206 and liberate the transcription of its target gene, ubiquitin-specific peptidase 33 (USP33). USP33, as a deubiquitinating enzyme, could deubiquitinate c-Myc, an important proliferation regulator (Soucek et al., 2008). These data demonstrate the role of circ_0057558/miR-206/USP33/c-Myc in prostate carcinogenesis.

## Materials and Methods

### Cell Culture and Prostate Cell Lines

Human 22RV1, DU145, PC3, and 293T cells were purchased from the Culture Collection of the Chinese Academy of Sciences (Shanghai, China). 22RV1 cells were grown in RPMI-1640 (Life Technologies, Grand Island, NY, United States), DU145 and PC3 cells were cultured in MEM (Life Technologies), while 293T cells were maintained in DMEM (Life Technologies). All media were supplemented with 10% fetal bovine serum (FBS) (GIBCO, Carlsbad, CA, United States). All cells were maintained at 37°C, 5% CO_2_.

### Prostate Cancer and Paracancerous Tissue Samples

Thirty-five patients admitted at Zhongshan Hospital Affiliated to Fudan University were enrolled in this study after written informed consent was provided. After the prostate cancer and paracancerous tissue specimens were resected during the surgery, the specimens were preserved and stored at −80C°. This study was in accordance with The Ethics Committee of Zhongshan Hospital Affiliated to Fudan University (Shanghai, China).

### Plasmid Construction

Plasmids for knocking down circ_0057558 (shcirc#1 and shcirc#2) and control shRNAs (shNC#1 and shNC#2) were constructed by inserting shRNAs-specific targeting circ_0057558 (Supplementary Table 1) and control shRNAs into a pLKO.1 vector (Addgene, Cambridge, MA, United States), respectively. Plasmids for the overexpression of circ_0057558 (circOE) and USP33 (USP33OE) were constructed by inserting the sequences of circ_0057558 and human USP33 into pcDNA3.1(+) CircRNA Mini Vector (Addgene, Cambridge, MA, United States) and pCDNA3.1(+) (Invitrogen, Carlsbad, CA, United States), respectively. pGL3-USP33 wild type (WT) was constructed by inserting the sequence of USP33 3′ untranslated region (UTR) into the pGL3-Promoter vector (Promega, Durham, NC, United States), and pGL3-USP33 mutant was generated by site-directed mutagenesis using the Quickchange Kit (Stratagene, La Jolla, CA, United States).

### Lentivirus Production

Lentivirus expressing circ_0057558 shRNAs and control shNC was produced by the co-transfection of lentivirus plasmids together with packaging plasmids into 293T cells with Lipofectamine 2000 (Invitrogen) as per manufacturer’s guideline. At 48-72 post-transfection, lentiviruses were collected from the medium and used to infect prostate cancer cells.

### Transfection of miR-206 Mimics, miR-206 Inhibitor, and USP33 siRNAs

miR-206 mimics (miR-mimics), miR-206 inhibitor (miR-inh), and negative control (miR-NC), as well as the siRNAs against USP33 (si#1 and si#2) and control siRNA (siNC) (Supplementary Table 2), were obtained from GenePharma (Shanghai, China) All transfections were performed using Lipofectamine 2000 (Invitrogen) as per the manufacturer’s instruction.

### Cell Proliferation Assay

Cells were plated at a density of 3 × 10^3^ cells per well in 96-well plates and treated as indicated in figure legends. After 0, 12, 24, and 48 h of treatment, Cell Count Kit-8 (CCK-8, SAB Biotech, College Park, MD, United States) reagent was added and incubated at 37°C, 5% CO_2_ for another 1 h. The optical density was recorded at 450 nm (OD 450) using a microplate reader.

### Colony Formation Assay

Cells cultured in 60-mm plates (1000 cells per dish) were treated as indicated in figure legends and cultured at 37°C, 5% CO_2_ until the colonies appeared. The medium was changed every 3 days during this period. Finally, after fixing with methanol, the cells were stained with 0.1% crystal violet.

### Analysis of Cell Cycle Distribution

After washing three times with phosphate-buffered saline (PBS), the collected cells were fixed with cold 80% ethanol at 4°C overnight. Then, the cells were incubated with 1 mg/ml RNase A and 0.1 mg/ml propidium iodide (PI) for 20 min in the dark at room temperature. The cell cycle distribution was evaluated by flow cytometry (BD Biosciences, San Jose, CA, United States).

### Quantitative Real-Time Polymerase Chain Reaction Analysis

Total RNA was isolated with Trizol reagent (Invitrogen) as per the manufacturer’s instructions. After cDNA was synthesized with Reverse Transcription Kit (Thermo Fisher Scientific, Rockford, IL, United States), quantitative real-time polymerase chain reaction (qRT-PCR) analysis was conducted to determine the expression of circRNA, mRNA, and miRNA with SYBR Green PCR Kit (Thermo Fisher Scientific) on the ABI 7500HT (Applied Biosystems, Foster City, CA, United States) to measure. All primers are listed in Supplementary Table 3.

### Western Blotting

Protein lysates were prepared from cultured cells with RIPA buffer containing protease inhibitors (Beyotime, Shanghai, China). After mixing with the sample buffer, the lysates were boiled for 5 min, separated by sodium dodecyl sulfate-polyacrylamide gel electrophoresis (SDS-PAGE), and blotted onto nitrocellulose membranes. Then, western blotting was done with the primary antibodies against USP33 (Abcam, Cambridge, MA, United States), c-Myc (Abcam), and GAPDH (Cell Signaling Technology, Danvers, MA, United States), followed by horseradish peroxidase (HRP)-linked secondary antibodies (Beyotime). The signals were developed by enhanced chemiluminescence (ECL) system (Millipore Biotech., Bredford, MA, United States).

### RNA Pull-Down Assay

Pull-down assay was performed with biotinylated-probe (RiboBio, Guangzhou, China), which was complemented to the junction area of circ_0057558 (5′-TATGTAGCCTTGGT GGATATGCCTGGATTTGTGGTATCATTT-3′). 22RV21 cells were lysed in lysis buffer containing RNase inhibitor (Promega, Madison, WI, United States). The cell lysates were incubated with probes for 2 h and then with Dynabeads^TM^ M-280 Streptavidin (Invitrogen) for 4 h. Subsequently, RNA was extracted from the by Trizol reagent and analyzed by qRT-PCR assay.

### Luciferase Reporter Assay

pGL3-USP33 and miR-206 mimics (miR-mimics) or control (miR-NC) was co-transfected into 22RV1 cells using Lipofectamine 2000 (Invitrogen). After cultured at 37°C, 5% CO_2_ for 48 h, Dual-Luciferase Reporter Assay System (Promega) was used to determine the luciferase activity and Renilla luciferase activity. The relative luciferase activity was normalized to the control group (miR-NC).

### RNA Immunoprecipitation

RNA immunoprecipitation assay was performed with Magna RIP^TM^ RNA-Binding Protein Immunoprecipitation Kit (Millipore Biotech). Cells were lysed with RIPA buffer containing RNase inhibitor (Promega). The cell lysates were incubated with RIP buffer containing magnetic beads conjugated with Argonaute 2 (Ago) antibody (Abcam) or IgG. After digesting with Dnase I and Proteinase K (Sigma-Aldrich, St. Louis, MO, United States), the immunoprecipitated RNA was isolated, and the enrichment of USP33 mRNA and miR-206 was detected by qRT-PCR.

### Immunoprecipitation

Total lysate was extracted from the indicated cells in RIPA buffer and incubated with anti-USP33 (Abcam), anti-c-Myc (Abcam), or control IgG (Santa Cruz) at 4°C. After 2 h, b protein A/G Plus agarose beads (Santa Cruz) was added and incubated at 4°C for another 2 h. Following washing four times with RIPA buffer, the protein complexes were mixed with sample buffer, boiled for 5 min, separated by SDS-PAGE, and finally subjected to western blot analysis with anti-USP33, anti-c-Myc, or anti-ubiquitin (Abcam).

### Tumor Formation *in vivo*

Animal experiments were approved by the Ethical Committee of Zhoupu Hospital Affiliated to Shanghai University of Medicine and Health Sciences (Shanghai, China). Four-week-old pathogen-free female BALB/c athymic nude mice were randomly divided into two groups (*n* = 18 per group). 22RV1 cells stably expressing circ_0057558 shRNA (shcirc#1) or control shRNA (shNC#1) in 0.1 ml PBS were subcutaneously injected into the nude mice (5 × 10^6^). Tumor length (L) and width (W) were examined every 3 days after the xenograft formation. Tumor volume (V) was calculated using the following equation: *V* = 0.5 × *L* × *W*^2^. At 33 days post-injection, six mice from each group were euthanized, and the subcutaneous tumors were weighed and subjected to imummuhistochemical analysis with anti-Ki-67. The survival rates of the remaining nude mice (*n* = 12 per group) were recorded for 90 days.

To evaluate the outcome of docetaxel treatment, 24 nude mice were subcutaneously injected with PC3 cells stably expressing circ_0057558 (cirOE) or control vector (5 × 10^6^ cells per mouse, *n* = 12 per cells). When the volume of xenograft reached 100 mm^3^, the mice were intraperitoneally administered with docetaxel (Jorfi et al., 2015) (10 mg/kg/day) or vehicle every 3 days. Twelve days after the treatment, the mice were sacrificed, and the subcutaneous tumors were weighed and subjected to imummuhistochemical analysis with anti-Ki-67.

### Isolation and Treatment of Primary Prostate Cancer Cells

Primary prostate cancer cells were isolated from 10 patients who underwent surgery at Zhongshan Hospital Affiliated to Fudan University. Written informed consent was taken from all participants. qRT-PCR was conducted to assess circ_0057558 expression in primary cells. The cells were plated onto 96-well plates, cultured overnight, and then treated with 6 μM MYCi361 (Selleck) (Han et al., 2019) or vehicle (DMSO). After 48 h of culture, CCK-8 assay was conducted as described above. The inhibition rate of cell proliferation was calculated as a percentage relative to vehicle control.

### Patient-Derived Xenograft

Patient-derived xenograft (PDX) model was established using mice in a NOD/SCID/IL2rg-/-(NSG) background (Jiangsu Biocytogen Co. Ltd, Nantong, China) to evaluate the anti-cancer activity of MYCi361 treatment. The 6-week-old immunodeficient mice (16–18 g) were housed under SPF condition (50–60% relative humidity, 12 h dark/light cycle) with free access to food and water. Fresh tumor tissues were collected from consenting prostate cancer patients admitted at Zhongshan Hospital Affiliated to Fudan University between August 2018 and April 2019. The tumor tissues from 10 patients were cut into 2 mm^3^ fragments (F0) and transplanted subcutaneously into the back of mice using a trocar gauge to generate F1 PDX tumors as previously described (Russell et al., 2015). When the grafts reached 5–10 mm in diameter, the expression of circ_0057558 in F1 PDX was detected by qRT-PCR and divided into circ0057558^high^ group and circ_0057558^low^ group. F1 PDXs were cut into 2 mm^3^ fragments and inoculated into NOD/SCID/IL2rg-/-(NSG) mice to generate F2 PDXs (*n* = 10 per group). Two weeks after inoculation, F2 PDX was successfully established, and the mice were treated with MYCi361 (55 mg/kg/day) (Han et al., 2019) or DMSO once a week by intraperitoneal injection. After 42 days from the first treatment, the mice were euthanized, and the tumors were harvested, weighed, and subjected to imummuhistochemical analysis with anti-Ki-67.

### Statistical Analysis

All *in vitro* experiments were repeated three times independently. All data were analyzed with Graphpad Prism software version 6.0 (GraphPad, San Diego, CA, United States). Student’s *t*-test was carried out to compare the statistical differences between the two groups, while one-way ANOVA followed by Tukey’s test was applied for more than two groups. *P* < 0.05 represents statistical significance.

## Results

### circ_0057558 Knockdown Suppressed Prostate Cancer Cell Proliferation and Induced Cell Arrest

**FIGURE 1:**
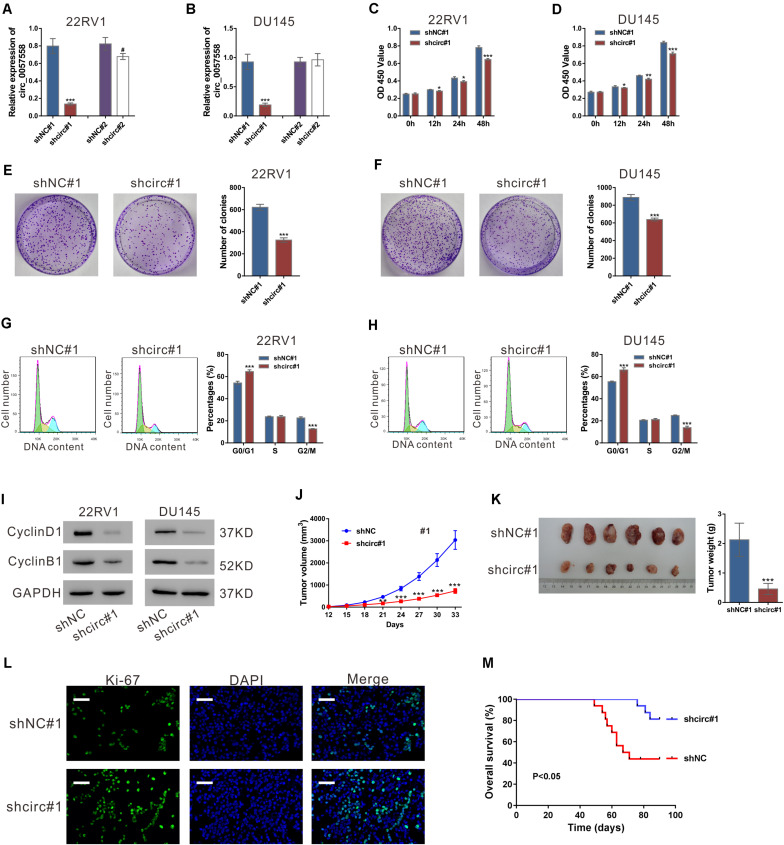
circ_0057558 knockdown suppressed prostate cancer cell proliferation *in vitro* and *in vivo*. **(A,B)** Lentivirus expressing specific shRNAs targeting circ_0057558 (shcirc#1 and shcir#2) and control shRNAs (shNC#1 and shNC#2) were infected into 22RV1 **(A)** and DU145 cells **(B)**. circ_0057558 expression was detected by qRT-PCR at 48 h post infection. Wild-type cells (WT) that had no treatment were used as negative control. **(C,D)** CCK-8 assay was carried out to detect proliferation in circ_0057558 knockdown group (shcirc#1) and control group (shNC#1). **(E,F)** Colony formation assay was conducted to determine the colony-forming ability of circ_0057558 knockdown group (shcirc#1) and control group (shNC#1). **(G,H)** Cell cycle analysis detected by PI staining and flow cytometry analysis. **(I)** CyclinD1 and CyclinB1 were detected by western blotting. **(J–M)** 22RV1 cells infected with circ_0057558 shRNA (shcirc#1) or control shRNA (shNC#1) were transplanted into nude mice (*n* = 6 per group). The tumor growth curves **(J)**, as well as the photos and weight **(K)** of xenografts on 33 days after inoculation are shown. Immunofluoresence staining with anti-Ki-67 **(L)** was carried out to assess cell proliferation in xenografts. Scale bar: 50 μm. **(M)** The Kaplan–Meier plot of survival duration in nude mice transplanted with 22RV1 inoculation infected with shcirc#1 or shNC#1 (*n* = 12 per group). Data were expressed as mean ± SD. *P < 0.05, **P < 0.01, ***P < 0.001 versus shNC#1; ^#^*P* < 0.05 versus shNC#2.

In our previous study, circ_0057558 expression has been identified to be elevated in prostate cancer tissues and cell lines (Xia et al., 2018). Lentivirus expressing specific shRNAs targeting circ_0057558 (shcirc#1 and shcir#2) were infected into 22RV1 and DU145, which expressed relative high level of circ_0057558 (Xia et al., 2018). shcirc#1 led to a significant reduction in circ_0057558 expression (Figures 1A,B) as compared to control shRNA (shNC#1), but showed little effect on the expression of its linear host mRNA (SLC39A10) (Supplementary Figure 1). shcirc#1 was used in the subsequent *in vitro* assays.

To investigate the cellular function of circ_0057558, CCK-8 assay, colony formation assay, and cell cycle analysis were then conducted. CCK-8 assay indicated that the proliferation rate of both the prostate cancer cell lines was significantly attenuated after circ_0057558 interference (Figures 1C,D). Colony formation assay indicated that circ_0057558 interference remarkably reduced the number of colonies, suggesting the role of circ_0057558 in the long-term proliferation of prostate cancer cells (Figures 1E,F). Cell cycle analysis showed that circ_0057558 knockdown notably increased the percentage of G0/G1 phase and decreased the percentage of G2/M phase compared to the control group (shNC#1) (Figures 1G,H). Moreover, the expression of cell-cycle regulators, CyclinD1 and CyclinB1, was also decreased by circ_0057558 interference (Figure 1I). Thus, results indicated that circ_0057558 knockdown suppressed prostate cancer cell cycle transition and proliferation *in vitro*.

To determine the effect of circ_0057558 on prostate cancer *in vivo*, we assessed the growth ability of 22RV1 cells with circ_0057558 knockdown in nude mice. The tumor growth curves demonstrated that the growth rate of xenografts formed from shcirc#1-infected cells was evidently slower than that from shNC#1-infected cells (Figure 1J). On day 33, the weight of the shcirc#1 xenografts was significantly lower than that of shNC#1 xenografts (Figure 1K). The ratio of Ki-67 positive cells was decreased in the shcirc#1 xenografts (Figure 1L). The Kaplan–Meier curves indicated that the nude mice treated with shcirc#1-infected cells had a longer overall survival time than those treated with shNC#1-infected cells (Figure 1M). These data indicated that circ_0057558 knockdown suppressed prostate cancer cell proliferation *in vivo*.

### circ _0057558 Expression Level Influenced the Sensitivity of Prostate Cancer Cells to Docetaxel

**FIGURE 2:**
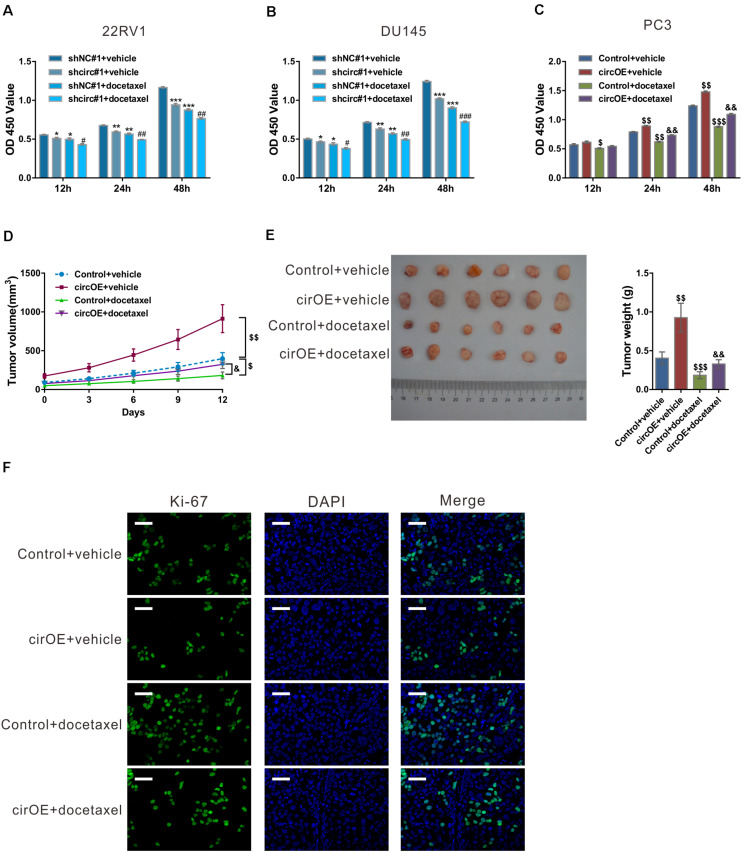
circ_0057558 expression level influenced the sensitivity of prostate cancer cells to docetaxel. **(A–C)** 22RV1 **(A)** and DU145 cells **(B)** were infected with lentivirus expressing specific shRNAs targeting circ_0057558 (shcirc#1) and control shRNAs (shNC#1), while PC3 cells were transfected with plasmid expressing circ_0057558 (circOE)/control vector (control). These cell lines were then exposed to 10 nM docetaxel or vehicle (DMSO). Cell proliferation was measured with CCK-8 assay. **(D–F)** Nude mice were divided into four groups (*n* = 6 per group) and subcutaneously injected with PC3 cells stably expressing circ_0057558 (cirOE) or control vector (5 × 10^6^ cells per mouse, *n* = 12 per cells). When the volume of xenograft reached 100 mm^3^, the mice were intraperitoneally administered with docetaxel (10 mg/kg/day) or vehicle every 3 days. The tumor growth curves **(D)**, as well as the photos and weight **(E)** of xenografts on 33 days after inoculation are shown. Immunofluoresence staining with anti-Ki-67 **(F)** was carried out to assess cell proliferation in xenografts. Scale bar: 50 μm. *P < 0.05, **P < 0.01, ***P < 0.001 versus shNC#1 + vehicle; ^#^*P* < 0.05, ^##^*P* < 0.01, ^###^*P* < 0.001 versus shNC#1 + docetaxel; ^$^*P* < 0.05, ^$$^*P* < 0.01, ^$$$^*P* < 0.001 versus control + vehicle; ^&^*P* < 0.05, ^&&^*P* < 0.01, ^&&&^*P* < 0.001 versus control + docetaxel.

We then explored whether circ_0057558 affected the efficacy of docetaxel in prostate cancer. CCK-8 results showed that docetaxel treatment suppressed the proliferation of 22RV1, DU145, and PC3 cells (Figures 2A–C). circ_0057558 knockdown strengthened the effects of docetaxel treatment in 22RV1 and DU145 cells (Figures 2A,B). Inversely, the overexpression of circ_0057558 remarkably weakened the effects of docetaxel treatment in PC3 cells (Figure 2C).

To evaluate the outcome of docetaxel treatment *in vivo*, xenograft model was established in nude mice by injection with PC3 cells stably expressing circ_0057558 (cirOE) or control vector, and the mice were then treated with docetaxel or vehicle. As shown in Figure 2D, xenografts overexpressing circ_0057558 had faster growth rate than those expressing control vector when docetaxel was applied. At 12 days after docetaxel treatment, the size and weight (Figure 2E) and cell proliferation (Figure 2F) were greater in docetaxel-treated xenografts overexpressing circ_0057558 than in those overexpressing vector. Together, these data suggested that the upregulated expression of circ_0057558 reduced the sensitivity of prostate cancer to docetaxel.

### circ_0057558 Sponged miR-206

**FIGURE 3:**
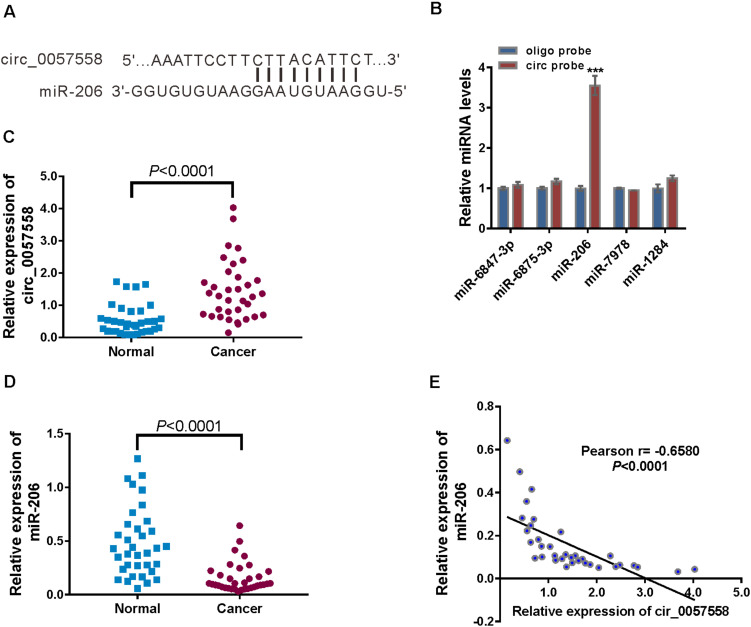
circ_0057558 was targeted by miR-206. **(A)** The putative binding sites of miR-206 and circ_0057558. **(B)** qRT-PCR analysis of the expression of candidate miRNAs in 22RV1 cells after biotin pull-down assay. ***P < 0.001 versus oligo probe. **(C,D)** Expression of circ_0057558 **(C)** and miR-206 **(D)** in prostate cancer tissue and adjacent non-tumor tissue. **(E)** Pearson *r* analysis showed the correlation between the expression of circ_0057558 and miR-206 in prostate cancer tissues.

Lately, accumulated evidence has indicated that circRNAs could absorb miRNAs, thereby reducing the regulation of miRNAs on their target genes (Qu et al., 2015). Several miRNAs were predicted to be associated with circ_0057558 by analysis with miRDB database^1^. The top rank miRNAs included hsa-miR-6847-3p, hsa-miR-6875-3p, hsa-miR-206, hsa-miR-7978, hsa-miR-206, and hsa-miR-1284. RNA pull-down analysis showed that only hsa-miR-206, which contains nine paired nucleotides with circ_0057558 (Figure 3A), was pulled down by circ_0057558 probe in 22RV1 cells (Figure 3B).

Further, significantly increased circ_0057558 expression (Figure 3C) and decreased miR-206 expression (Figure 3D) were detected in the prostate cancer tissues in comparison to the adjacent non-cancerous tissue. Moreover, miR-206 expression in prostate cancer tissues was negatively correlated with circ_0057558 expression (Figure 3E). Taken together, results indicate that miR-126 was targeted by circ_0057558.

### miR-206 Inhibitor Rescued the Function of circ_0057558 Knockdown, While miR-206 Mimics Rescued the Function of circ_0057558 Overexpression

**FIGURE 4:**
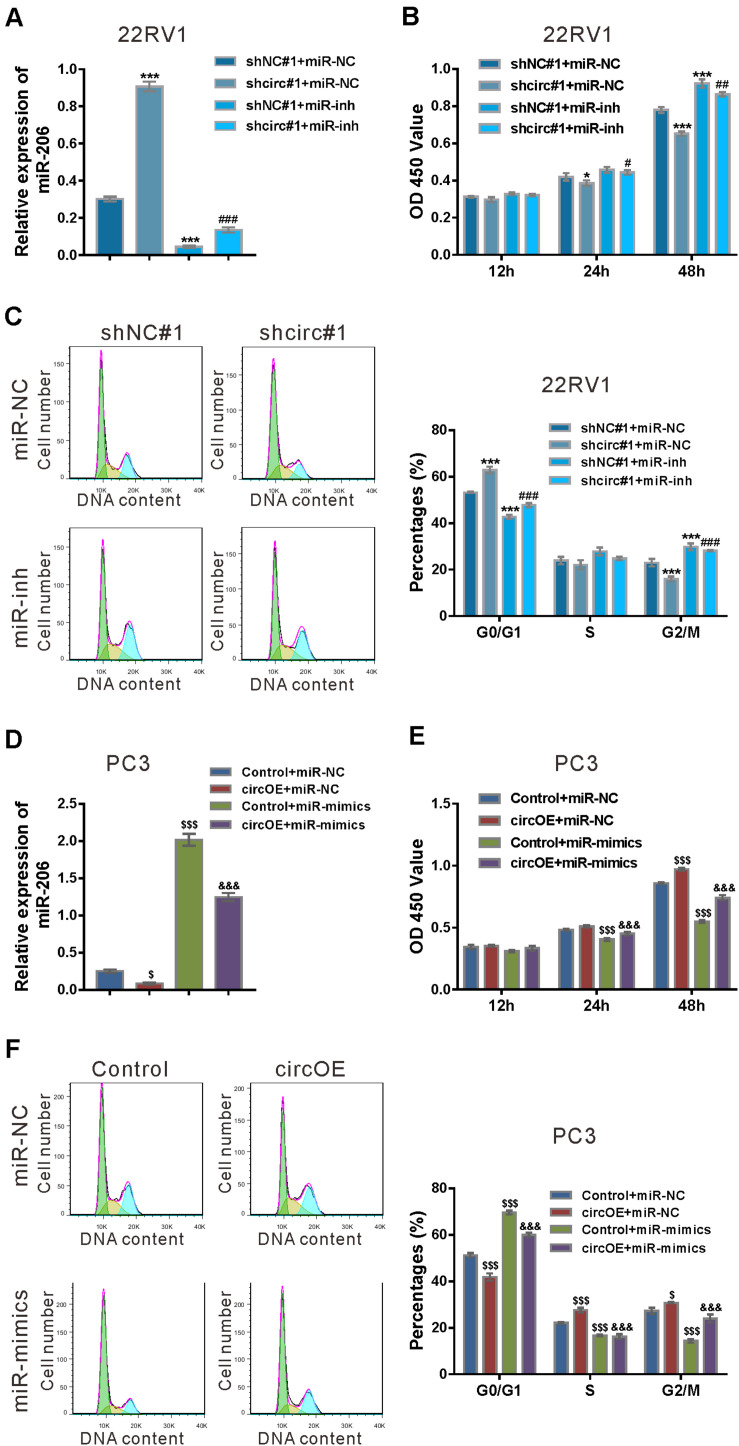
miR-206 inhibitor rescued the function of circ_0057558 knockdown, while miR-206 mimics rescued the function of circ_0057558 overexpression. **(A–C)** 22RV1 cells were transduced with lentivirus expressing circ_0057558 shRNA (shcirc#1)/control shRNA (shNC#1) and transfected with miR-206 inhibitor (miR-inh)/control (miR-NC). miR-206 expression **(A)** was detected by qRT-PCR at 48 h post treatment. CCK-8 assay **(B)** and cell cycle analysis **(C)** was carried out to detect cell proliferation and cell cycle distribution, respectively. *P < 0.05, ***P < 0.001 versus shNC#1 + miR-NC; ^#^*P* < 0.05, ^##^*P* < 0.01, ^###^*P* < 0.001 versus shcirc#1 + miR-NC. **(D–F)** PC3 cells were transfected with plasmid expressing circ_0057558 (circOE)/control vector (control) and transfected with miR-206 mimics (miR-mimics)/control (miR-NC). qRT-PCR **(D)**, CCK-8 assay **(E)**, and cell cycle analysis **(F)** were done to detect miR-206 expression, cell proliferation, and cell cycle distribution, respectively. ^$^*P* < 0.05, ^$$$^*P* < 0.001 versus control + miR-NC; ^&&&^*P* < 0.001 versus circOE + miR-NC.

To verify specific targeting of miR-206, 22RV1 cells were treated with lentivirus expressing circ_0057558 shRNA (shcirc#1)/control shRNA (shNC#1) and miR-206 inhibitor (miR-inh)/control (miR-NC). miR-206 inhibitor (miR-inh) significantly reduced miR-206 expression in cells infected with shcirc#1 or shNC#1 (Figure 4A). CCK-8 assay demonstrated that miR-206 inhibitor rescued the suppressed proliferation in shcirc#1-infected 22RV1 cells (Figure 4B). Moreover, cycle analysis assay revealed that miR-206 inhibitor reversed the role of circ_0057558 shRNA in the cell cycle arrest (Figure 4C).

Rescue experiments were also done in PC3 cells, which expressed relative low level of circ_0057558 (Xia et al., 2018). Lentivirus expressing circ_0057558 (circOE) profoundly increased circ_0057558 expression and did not affect SLC39A10 expression in PC3 cells as compared to the control vector (Vector) (Supplementary Figure 2). miR-206 mimics (miR-mimics) significantly enhanced miR-206 expression in cells infected with circOE or control (Figure 4D). CCK-8 assay (Figure 4E) and cell cycle analysis (Figure 4F) showed that miR-206 mimics inverted the effects of circ_0057558 overexpression on cell proliferation and cell cycle transition, respectively. Collectively, these data demonstrated that the function of circ_0057558 on prostate cancer cells was mediated by miR-206.

### circ_0057558 Regulated the miR-206 Target, USP33

**FIGURE 5:**
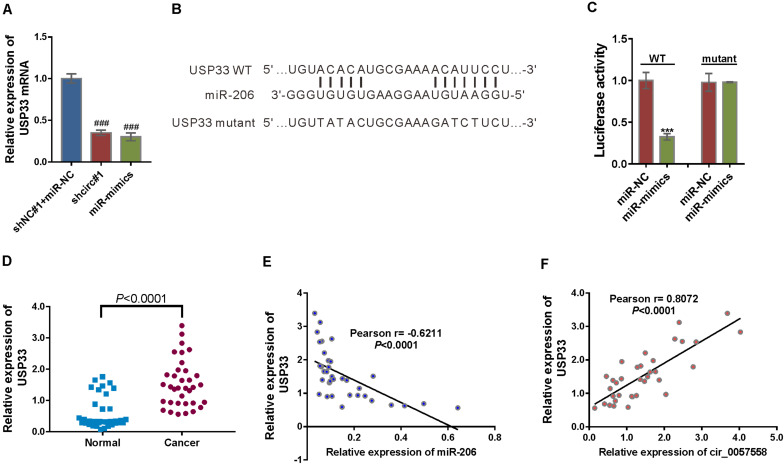
circ_0057558 regulated the miR-206 target, USP33. **(A)** mRNA expression of USP33 in 22RV cells infected with lentivirus expressing circ_0057558 shRNA (shcirc#1) or transfected with miR-206 mimics (miR-mimics). Cells infected with control shRNA (shNC#1) and transfected with control miRNA (miR-NC) were served as control. ^###^*P* < 0.001 versus shNC#1 + miR-NC. **(B)** The putative miRNA binding sites in the USP33 3′UTR. **(C)** pGL3-USP33 wild-type (WT) or mutant was co-transfected into 22RV1 cells with miR-206 mimics (miR-mimics) or control (miR-NC). Luciferase assay was performed to determine luciferase activity. The relative luciferase activity normalized to the control group (miR-NC). ***P < 0.001 versus miR-NC. **(D)** qRT-PCR analysis following the RIP assay was conducted to confirm the interaction between miR-206 with USP33. $$$*P* < 0.001 versus IgG. **(E)** Expression of USP33 mRNA in prostate cancer tissue and adjacent non-tumor tissue. **(F,G)** Pearson *r* analysis showed the correlation between the expression of miR-206 and USP33 and the expression of circ_0057558 and USP33 in prostate cancer tissues.

Among the putative target genes of miR-206 as predicted by miRDB, USP33, which functions in breast, lung, and colon cancer cells (Yuasa-Kawada et al., 2009; Wen et al., 2014; Huang et al., 2015; Liu et al., 2016), was selected. miR-206 mimics suppressed the mRNA expression of USP33 in 22RV1 cells, while miR-206 inhibitor had reverse effect (Figure 5A). To study whether miR-206 interacts with USP33, 22RV1 cells were transfected with reporter plasmid pGL3-USP33 WT, which contains the predicted binding site of miR-206, or pGL3-USP33 mutant, which contains mutations in the predicted binding site (Figure 5B). Luciferase assay showed that miR-206 mimics repressed the luciferase activity of pGL3-USP33 WT, while miR-206 mimics had little effect on that of pGL3-USP33 mutant (Figure 5C). RIP analysis followed by qRT-PCR assay demonstrated that USP33 mRNA and miR-206 were immunoprecipitated by Ago2 (Figure 5D), which confirmed the interaction between miR-206 and USP33.

Then USP33 mRNA expression was assessed in prostate cancer specimens and controls. USP33 mRNA expression was significantly increased in prostate cancer tissues compared to non-cancerous tissue samples (Figure 5E). Moreover, USP33 mRNA expression in prostate cancer tissues showed a negative correlation with miR-206 expression (Figure 5F) and a positive correlation with circ_0057558 expression (Figure 5G). Together, the findings proved that USP33 was a direct target gene of miR-206 in prostate cancer.

### USP33 Overexpression Rescued the Function of miR-206 Mimics

**FIGURE 6:**
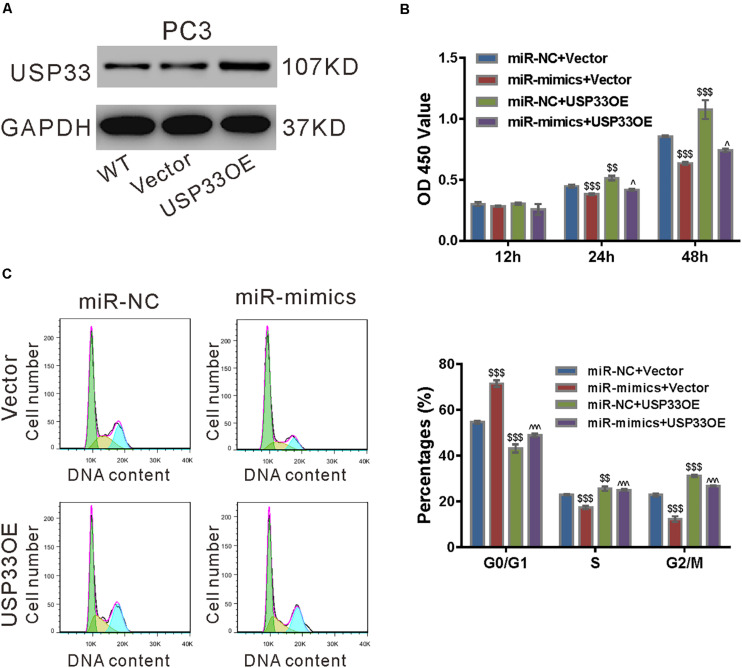
USP33 overexpression rescued the function of miR-206 mimics. **(A)** Protein expression of USP33 in PC3 cells infected with lentivirus expressing USP33 (USP33OE)/control vector (Vector). Representative blots from three independent experiments are shown. **(B–D)** PC3 cells were transfected with USP33OE/vector and transfected with miR-206 mimics (miR-mimics)/control (miR-NC). CCK-8 assay **(B)** and cell cycle analysis **(C)** were done to detect cell proliferation and cell cycle distribution, respectively. **(D)** CyclinD1 and CyclinB1 were detected by western blotting. **P < 0.01, ***P < 0.001 versus miR-NC + vector; ^#^*P* < 0.05, ^###^*P* < 0.001 versus miR-mimics + vector.

To ascertain the roles of USP33 in the function of miR-206, we determined if USP33 overexpression was able to rescue the effect of miR-206 mimics. As shown in Figure 6A, lentivirus expressing USP33 obviously increased its protein expression in PC3 cells. CCK-8 assay (Figure 6B), cell cycle analysis (Figure 6C), and expression of CyclinD1 and CyclinB1 (Figure 6D) showed that USP33 overexpression partially blocked the effects of miR-206 mimics on cell proliferation and cell cycle transition. These data suggest that the anti-proliferation effects of miR-206 may be mediated by the inhibition of USP33.

### USP33 Deubiquitinated c-Myc

As circ_0057558 functioned in prostate cancer cell cycle progression, we then detected the protein levels of several cell-cycle-related proteins in 22RV1 cells with circ_0057558 knockdown (Supplementary Figure 3A). The results showed that circ_0057558 knockdown decreased c-Myc protein expression, and qRT-PCR analysis revealed that circ_0057558 knockdown had little effect on c-Myc mRNA expression (Supplementary Figure 3B).

**FIGURE 7:**
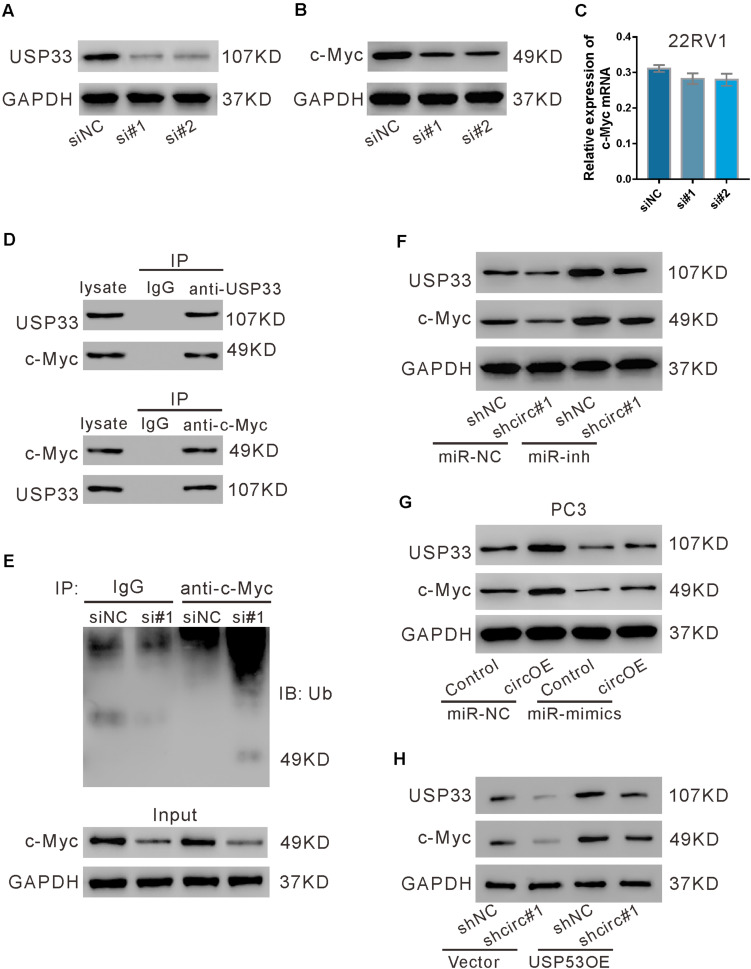
USP33 deubiquitinated c-Myc. **(A–C)** 22RV1 cells were transfected with USP33 siRNAs (si#1 and si#2) or control siRNA (siNC). After 48 h, the protein expression of USP33 **(A)** and c-Myc **(B)** and the mRNA expression of c-Myc **(C)** were evaluated. **(D)** 22RV1 cell lysate was immunoprecipitated (IP) with anti-USP33 (upper) or anti-c-Myc (lower) or control IgG and then immunoblotted (IB) with anti-USP33 and anti-c-Myc. **(E)** 22RV1 cells were transfected with USP33 siRNA (si#1) or siNC. Immunoprecipitation experiment was performed with anti-c-Myc or control IgG and IB with anti-ubiquitin (Ub). **(F)** 22RV1 cells were transduced with lentivirus expressing circ_0057558 shRNA (shcirc#1)/control shRNA (shNC#1) and transfected with miR-206 inhibitor (miR-inh)/control (miR-NC). The expression of USP33 and c-Myc was detected at 48 h post treatment. **(G)** PC3 cells were transfected with plasmid expressing circ_0057558 (circOE)/control vector (control) and miR-206 mimics (miR-mimics)/control (miR-NC). The expression of USP33 and c-Myc was assessed at 48 h post treatment. **(H)** 22RV1 cells were transduced with shcirc#1/shNC#1 and USP33OE/vector. The expression of USP33 and c-Myc was detected at 48 h post treatment.

We then investigated whether USP33 affected the expression of c-Myc in 22RV1 cells. As shown in Figure 7A, USP33 siRNAs (si#1 and si#2) obviously suppressed its protein expression. USP33 knockdown significantly reduced c-Myc protein expression (Figure 7B), but did not affect c-Myc mRNA expression (Figure 7C). Given that USP33 is a deubiquitinating enzyme, we speculated that USP33 regulated c-Myc protein through ubiquitination pathway. IP experiments showed that USP33 interacted with c-Myc (Figure 7D), and USP33 knockdown profoundly enhanced c-Myc ubiquitination (Figure 7E). These data suggested that USP33 may bind and deubiquitinate c-Myc in 22RV1 cells.

Further, c-Myc protein expression was reduced by circ_0057558 knockdown (Figure 7F), but was enhanced by circ_0057558 overexpression (Figure 7G). miR-206 inhibitor and miR-206 mimics reversed the effects of circ_0057558 knockdown and overexpression, respectively. Additionally, USP33 overexpression reversed the inhibitory effects of circ_0057558 knockdown on c-Myc expression (Figure 7H). These data suggested that circ_0057558 regulated c-Myc via miR-206/USP33.

### circ_0057558 Expression Level Was Correlated With the Proliferation Inhibition Effect of c-Myc Inhibitor MYCi361

**FIGURE 8:**
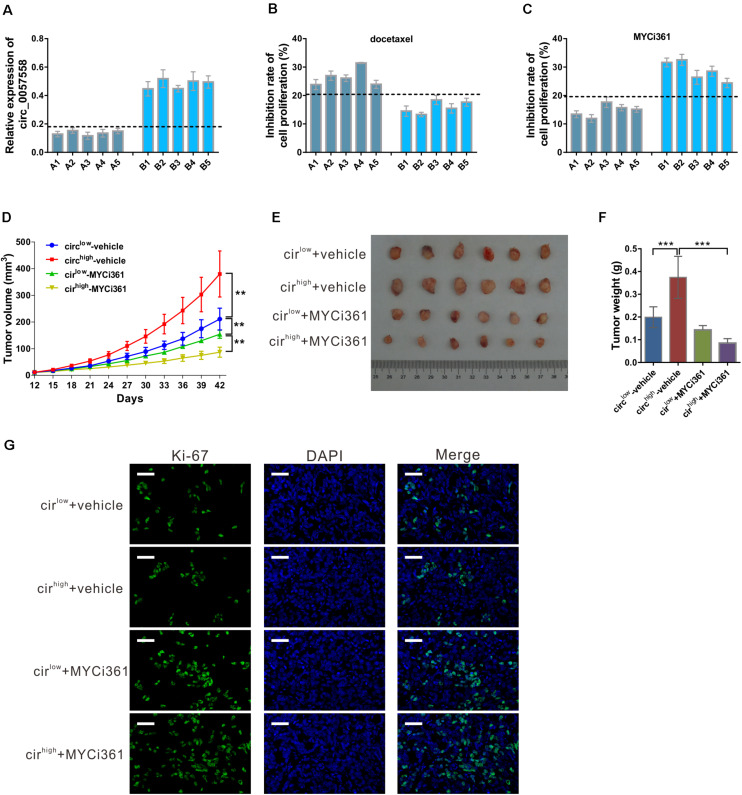
circ_0057558 expression level was correlated with the proliferation inhibition effect of c-Myc inhibitor MYCi361. **(A–C)** Primary prostate cancer cells were isolated as described in Materials and Methods section. circ_0057558 expression **(A)** in the primary prostate cancer cells was assessed by qRT-PCR. Primary cells were treated with 10 nM docetaxel **(B)**, 6 μM MYCi361 **(C),** or vehicle (DMSO) for 48 h. Inhibition rate of cell proliferation (%) was determined using CCK-8 assay. **(D–G)** Patient-derived xenograft (PDX) model was established with fresh prostate cancer tissues and defined as circ_0057558^high^ group and circ_0057558^low^ group. The F2 PDXs were treated with MYCi361 (55 mg/kg/day) and vehicle (DMSO) twice a week by intraperitoneal injection. Tumor growth curves **(D)** and the photos and weight of PDX **(E)** are shown. **(F)** Immunofluoresence staining with anti-Ki-67 was carried out to assess cell proliferation in xenografts. Scale bar: 50 μm. **P < 0.01, ***P < 0.001.

Previous evidence has shown that c-Myc inhibitor MYCi361 possesses anti-tumor activity both *in vitro* and *in vivo* (Han et al., 2019). Considering the positive regulatory role of circ_0057558 on c-Myc in prostate cancer, we supposed that circ_0057558 expression level influences the anti-tumor effect of MYCi361. To test this hypothesis, primary prostate cancer cells were isolated, defined as circ_0057558-low expression (A1–A5) group and circ_0057558-high expression (B1–B5) group (Figure 8A), and treated with docetaxel, MYCi361, or vehicle for 48 h. The results showed that docetaxel exposure-induced cell proliferation inhibition was more aggravated in prostate cancer cells with lower expression levels of circ_0057558 (A1–A5) than in those with higher expression levels of circ_0057558 (B1-B5) (Figure 8B), while MYCi361 showed reverse effects (Figure 8C).

Additional, PDX model was established and then treated with MYCi361. These xenografts were divided as circ_0057558^high^ group and circ_0057558^low^ group. As shown in Figure 8D, circ_0057558^high^ xenografts treated with vehicle grew the fastest, while circ_0057558^high^ xenografts treated with MYCi361 grew the slowest. At the end of the experiment, MYCi361-treated circ_0057558^high^ xenografts showed significant smaller tumor size and weight than vehicle-treated circ_0057558^high^ xenografts, while the change in MYCi361-treated circ_0057558^low^ group was not significant compared to the vehicle-treated circ_0057558^low^ group (Figures 8E,F). The changes of cell proliferation in xenografts as indicated by immunofluorescence staining with anti-Ki-67 followed the same trend (Figure 8G). Therefore, the efficacy of MYCi361 was more evident in the treatment of circ_0057558^high^ xenografts than in circ_0057558^low^ xenografts.

## Discussion

circRNAs were firstly found in viruses in 1970s (Sanger et al., 1976) and later in human cell lines and the human body (Cocquerelle et al., 1993). Recent evidence has revealed that the dysregulated expression of circRNAs plays an important role in tumorigenesis (Rong et al., 2017; Kristensen et al., 2018; Zhong et al., 2018). Previously, we have reported that circ_0057558 was upregulated in prostate cancer tissues and cells (Xia et al., 2018). The present study was aimed to determine the biological function of circ_0057558 in prostate cancer. *In vitro* cellular experiments indicated that circ_0057558 silencing induced by circRNA-specific shRNA could significantly repress the proliferation and docetaxel resistance and induce cell-cycle arrest at G0/G1 phase. *In vivo* xenograft experiments revealed that tumor growth was suppressed by circ_0057558 silencing and that the nude mice treated with circ_0057558 silencing cells had a longer overall survival time. Upregulated expression of circ_0057558 reduced the sensitivity of xenograft to docetaxel. These data suggest that circ_0057558 plays an oncogenic role in prostate cancer.

Since circ_0057558 could promote prostate cell proliferation, we then tried to figure out how it took part in the prostate carcinogenesis. Numerous evidence has showed that circRNAs serve as a miRNA sponge during tumorigenesis (Zheng et al., 2016; Tang et al., 2017; Xu et al., 2017; Zhu et al., 2017; Pan et al., 2018). We hypothesized that circ_0057558 might sequester miRNAs thereby inducing the transcription of their target genes. In order to support this view, the possible downstream miRNAs of circ_0057558 were predicted by bioinformatics analysis. To validate the miRNAs regulated by circ_0057558, RNA-pull down assay was performed, and hsa-miR-206 was pulled down by circ_0057558 probe in prostate cancer cells. In prostate cancer tissues, miR-206 expression was negatively correlated with circ_0057558 expression. These data indicate the regulation role of circ_0057558 on miR-206. Earlier study has reported that miR-206 may act as a skeletal muscle specific miRNA involved in skeletal muscle differentiation (Anderson et al., 2006). Currently, several studies have shown that miR-206 is a negative regulator in human cancer progression including prostate cancer (Yan et al., 2009; Chen et al., 2015; Dai et al., 2018; Wang et al., 2018). Here, miR-206 was also found to negatively regulate prostate cancer cell proliferation. Lower expression of miR-206 by inhibitor rescued the function of circ_0057558 knockdown, whereas higher expression of miR-206 by mimics rescued the function of circ_0057558 overexpression. Taken together, these results demonstrated that circ_0057558 influences cell cycle progression in prostate cancer cells through miR-206.

Then we analyzed the possible target genes of miR-206. The bioinformatics analysis, luciferase assay, and RIP assay identified USP33 as a target gene of miR-206. USP33 plays an important role in breast, lung. and colon cancer (Yuasa-Kawada et al., 2009; Wen et al., 2014; Huang et al., 2015; Liu et al., 2016). In our study, we found that USP33 overexpression partially reversed the negative regulated effects of miR-206 mimics on prostate cell proliferation. Moreover, the suppression of circ_0057558 reduced the mRNA expression of USP33. Moreover, qRT-PCR revealed that USP33 was upregulated in prostate cancer tissues. USP33 mRNA expression in prostate cancer tissues has negative correlation with miR-206 expression and positive correlation with circ_0057558 expression. Further, circ_0057558 knockdown reduced protein levels of c-Myc and had no effects on its mRNA levels. c-Myc, a well-known proliferative regulator, acts as an oncoprotein in diverse human cancers (Soucek et al., 2008). IP experiments revealed that USP33 could bind and deubiquitinate c-Myc. Decreased c-Myc protein expression by circ_0057558 knockdown was partially reversed by miR-206 inhibitor. All these findings propose that miR-206/USP33/c-Myc axis may mediate the oncogenic role of circ_0057558 in prostate cancer.

Moreover, c-Myc inhibitors have shown powerful anti-cancer potentials in prostate cancer (Han et al., 2019). When c-Myc inhibitor MYCi361 was applied *in vitro* and *in vivo*, better tumor suppressing effects were observed in circ_0057558^high^ groups. Our data suggested that the impact of circ_0057558 expression should be taken into account before the anti-tumor therapies targeting c-Myc are applied to prostate cancer patients.

In addition, there are some limitations associated with the current study. First, the sample size of tumor sample is relatively small. Second, immunohistochemistry (IHC) staining of USP33 and c-Myc, as well as *in situ* hybridization (ISH) of circ_0057558 and miR-206, was not included. IHC and ISH on a large cohort of prostate cancer samples will be performed in the future, which will strength our current findings.

Collectively, the current study shows that circ_0057558 gives an impetus to cell proliferation and cell cycle transition in prostate cancer cell lines by sponging miR-206 and positively regulating the transcription of the miR-206 target gene USP33. These findings may provide a novel insight for prostate cancer therapy.

## Data Availability Statement

The original contributions presented in the study are included in the article/Supplementary Material, further inquiries can be directed to the corresponding author/s.

## Ethics Statement

The studies involving human participants were reviewed and approved by The Ethics Committee of Zhongshan Hospital Affiliated to Fudan University (Shanghai, China). The patients/participants provided their written informed consent to participate in this study. The animal experiment protocol was approved by the Ethical Committee of Zhoupu Hospital Affiliated to Shanghai University of Medicine and Health Sciences (Shanghai, China).

## Author Contributions

TD and JZ contributed to the study design. TD, YZ, HJ, and PZ conducted the study. TD, YZ, and JG contributed to the data collection and analysis. TD and YZ drafted the manuscript. TD and JZ revised the manuscript. All authors approved the final version of the manuscript for submission.

## Conflict of Interest

The authors declare that the research was conducted in the absence of any commercial or financial relationships that could be construed as a potential conflict of interest.

## Footnotes

1 http://mirdb.org/miRDB/index.html
