# Supplementary material for: Circular RNA circ_0057558 Controls Prostate Cancer Cell Proliferation Through Regulating miR-206/USP33/c-Myc Axis
Source: Front Cell Dev Biol. 2021 Feb 26;9:644397. doi: 10.3389/fcell.2021.644397 (PMC7952531; doi:10.3389/fcell.2021.644397)
Supplement: Supplementary file 1 [file Table_1.DOCX]

**Table S1.** Target sequence of circ_0057558 shRNAs.

| Name | Target sequence |
| --- | --- |
| shNC#1 | 5’- GAACCACCTATACCCTGGA-3’ |
| shcirc#1 | 5’- GAACCACCTATACGGACCT -3’ |
| shNC#2 | 5’- GATATGGGACCTAAACACC-3’ |
| shcirc#2 | 5’- CTATACGGACCTAAACACC-3’ |

**Table S2.** Sequences of miRNA and siRNA related sequence.

| Name | Sequence |
| --- | --- |
| miR-206 mimics (miR-mimics) | 5’-UGGAAUGUAAGGAAGUGUGUGG-3’ |
| miR-206 inhibitor (miR-inh) | 5’-CCACACACUUCCUUACAUUCCA-3’ |
| Control miRNA (miR-NC) | 5’-CAGUACUUUUGUGUAGUACAA-3’ |
| USP33 siRNA (si#1) | 5’- GGACCAAAUCUUUGGGCAUUU -3’ |
| USP33 siRNA (si#2) | 5’- GCCUACUACUCUGUUUCAAUU -3’ |
| Control siRNA (siNC) | 5’- CAGUACUUUUGUGUAGUACAA -3’ |

**Table S3.** Primer sequences for qRT-PCR.

| Name | Primers |
| --- | --- |
| USP33 | Forward: 5’- ATCTGTTGTGCCTACTACTC -3’  Reverse: 5’- GCTCTTGTCTTCTTCCATTG-3’ |
| c-Myc | Forward: 5’-CCTTCTTTCCTCCACTCTC -3’  Reverse: 5’- CAAACCCTCTCCCTTTCTC-3’ |
| GAPDH | Forward: 5’- AATCCCATCACCATCTTC -3’  Reverse: 5’- AGGCTGTTGTCATACTTC -3’ |
| SLC39A10 | Forward: 5’- ACCACCACGGCGAGAAC-3’  Reverse: 5’- GTCAATCCAGCACTGAAAGC-3’ |
| circ_0057558 | Forward: 5’-AGTCACTGCAGGCATGTTC -3’  Reverse: 5’-TGCAACAAGGAATGTAAGA-3’  Probe: 5’- FAM-TTGGTGGATATGCCTGGATTTGTGG-BHQ1-3’ |
| miR-206 | RT-primer: 5’- GTCGTATCCAGTGCAGGGTCCGAGGT  ATTCGCACTGGATACGACCACTTG-3’  Forward: 5’- GTAAGGAAGTGTGTGGTTTCGG -3’  Reverse: 5’- AGTGCAGGGTCCGAGGTATT -3’ |
| RNU6-1 | Forward: 5’- CTCGCTTCGGCAGCACA -3’  Reverse: 5’- AACGCTTCACGAATTTGCGT -3’ |


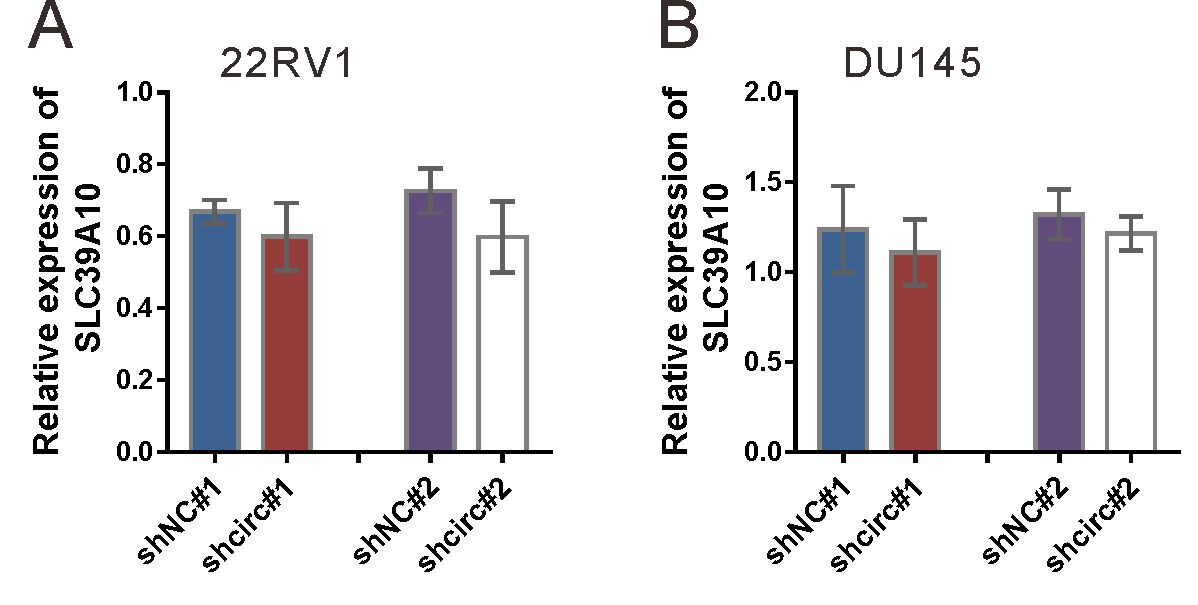


**Figure S1.** Effects of circ_0057558 knockdown on SLC39A10 expression. Lentivirus expressing specific shRNAs targeting circ_0057558 (shcirc#1 and shcir#2) and control shRNAs (shNC#1 and shNC#2) were infected into 22RV1 (A) and DU145 cells (B). SLC39A10 expression was detected by qRT-PCR at 48 h post infection. Wild-type cells (WT) without any treatment were served as negative control.


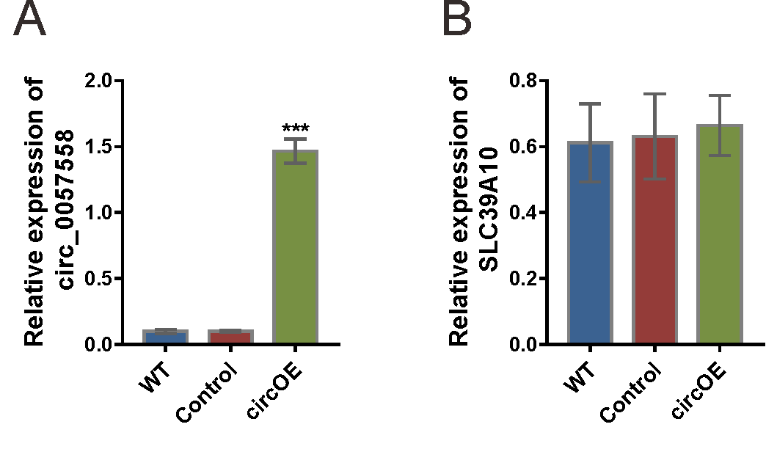


**Figure S2.** Effects of circ_0057558 overexpression on SLC39A10 expression. Relative expression of circ_0057558 (A) and SLC39A10 (B) in PC3 cells infected with lentivirus expressing circ_00577558 (circOE) and control vector (Control). Cells without any treatment were served as negative control (WT). ***P<0.01 vs Control.


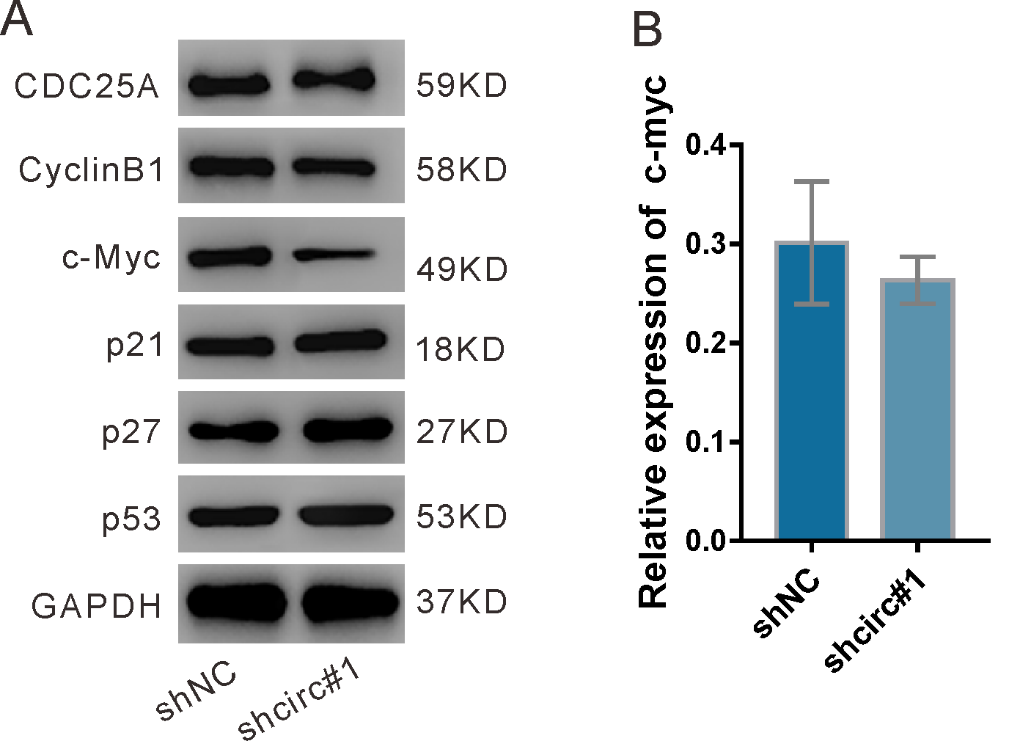


**Figure S3.** Effects of circ_0057558 knockdown on the expression of several cell-cylce-related proteins. 22RV1 cells were infected with lentivirus expressing circ_0057558 shRNA (shcirc#1) or control shRNA (shNC). (A) Protein expression of cell-cycle related proteins was detected. (B) mRNA expression of c-Myc was assessed.
